# A novel optical flow method for myocardial deformation analysis from tagged MRI

**DOI:** 10.1186/1532-429X-14-S1-W45

**Published:** 2012-02-01

**Authors:** Mohammadreza Negahdar, Hui Wang, Amir Amini

**Affiliations:** 1 Electrical and Computer Engineering, University of Louisville, Louisville, KY, USA

## Background

Cardiac motion analysis can play an important role in diagnosis of cardiac disease. Tagged magnetic resonance imaging (MRI) has the ability to directly and non-invasively alter tissue magnetization and produce tag patterns on the deforming tissue [1-3]. This abstract proposes a novel optical flow method for computing the left ventricular systolic dynamics using harmonic phase (HARP) [4] images extracted from tagged MRI data.

## Methods

Tagged MRI gives rise to spectral peaks in k-space, each peak containing information about motion in a particular direction. Harmonic images are produced by filtering the spectral peaks in the Fourier domain and extracting the spatial phase information from the inverse Fourier transform of the filtered images [4,5].

The horizontal and vertical components of the optical flow field are jointly estimated from harmonic phase images in the two directions. The basic assumption in standard optical flow estimation is the grey value constancy. In the proposed approach, we additionally include gradient constancy and mass conservation which are applied to the image globally, and spatio-temporal smoothness which is applied in a local fashion. For more details, please refer to [6].

## Results

**Figure 1:**
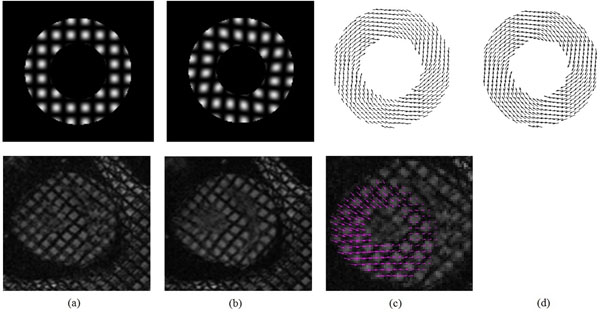
Tagged MR images and computed motion field between two frames for simulated data (row 1) and canine data (row 2). (a) reference image, (b) deformed image, (c) motion field between reference image and deformed image as computed with the proposed method, (d) ground truth motion field for simulated data.

Our method has been applied to both simulated data and in vivo canine data. Two frames of simulated data and two frames of a canine study together with the computed motion fields are shown in Figure 1. Table 1 reports the angular error (AE) and relative root mean squared (RRMS) error between the calculated and the ground-truth motion fields with SinMod [5] and the proposed method.

**Table 1:** AE and RRMS error between the ground truth motion field (obtained from the cardiac motion simulator) and motion fields calculated with SinMod and the proposed method.

Frame Numbers	SinMod		Proposed Method	
	AE	RRMS (%)	AE	RRMS (%)

Frame 1-2	9.63°	15.40	7.62°	14.45
Frame 1-3	8.55°	15.91	5.99°	12.92
Frame 1-4	7.93°	15.35	6.56°	13.66
Frame 1-5	8.66°	15.43	6.90°	13.75

## Conclusions

We have presented a novel optical flow method for cardiac motion tracking. Four physical constraints were incorporated into the optical flow energy function to obtain a robust and accurate motion field.
